# Correlations between periparturient serum concentrations of non-esterified fatty acids, beta-hydroxybutyric acid, bilirubin, and urea and the occurrence of clinical and subclinical postpartum bovine endometritis

**DOI:** 10.1186/1746-6148-6-47

**Published:** 2010-10-27

**Authors:** Toschi B Kaufmann, Marc Drillich, Bernd-Alois Tenhagen, Wolfgang Heuwieser

**Affiliations:** 1 Clinic for Animal Reproduction, Section of Production Medicine and Quality Management, Freie Universität Berlin, Berlin, Germany; 2 Clinic for Ruminants, Section for Herd Health Management, Vetmeduni Vienna, Vienna, Austria; 3 Federal Institute for Risk Assessment (BfR), Section Biological Safety, Berlin, Germany

## Abstract

**Background:**

Postpartum endometritis in cattle is a multifactorial disease with high economic impact. Both, clinical endometritis (CE) and subclinical endometritis (SCE) result in decreased reproductive performance. Results from in vitro studies led to the implication that non-esterified fatty acids (NEFA), beta-hydroxybutyric acid (BHBA), bilirubin, and urea could be used as predictors for endometritis in veterinary practice. In this field study, we set out to establish optimal predictor cut points of these metabolic parameters for the detection of CE and SCE. Serum samples were collected one week prior to parturition (wk -1), in the first week postpartum (wk +1) and between 28 and 35 days postpartum (wk +5) from 209 Holstein-Friesian cows. At wk +5, all cows were examined for signs of CE and SCE.

**Results:**

Higher concentrations of urea at wk +1 were associated with increased odds of CE (OR = 1.7, P = 0.04) in primiparous (PP) cows. A predictor cut point of 3.9 mmol/L (sensitivity: 61%, specificity: 70%) was determined. In multiparous (MP) cows, the logistic regression model revealed that higher concentrations of NEFA at wk -1 were associated with increased odds of CE and SCE (healthy vs. CE: OR = 9.1, P = 0.05; healthy vs. SCE: OR = 12.1, P = 0.04). A predictor cut point of 0.3 mmol/L (sensitivity: 38%, specificity: 87% and sensitivity: 35%, specificity: 89%, respectively) was determined. Increasing concentrations of urea at wk +5 were associated with decreased odds of CE (healthy vs. CE: OR = 0.6, P = 0.01; SCE vs. CE: OR = 0.5, P = 0.03). A predictor cut point of 3.8 mmol/L (sensitivity: 52%, specificity: 81%) was determined. For BHBA and bilirubin relationships with CE or SCE were not detected.

**Conclusions:**

The corresponding combinations of sensitivity and specificity of the determined predictor cut points were not satisfactory for practical use. Thus, the analysed parameters, i.e. NEFA, BHBA, bilirubin, and urea, at the chosen time points, i.e. at wk -1, at wk +1, and at wk +5 relative to calving, are unsatisfactory for disease prediction. Further research is required to clarify the questions raised by the current study.

## Background

Postpartum endometritis in cattle is a multifactorial disease with high economic impact. Inflammation of the bovine uterus has been demonstrated to decrease reproductive performance. Both, clinical (CE) and subclinical endometritis (SCE) were associated with increased days to first service as well as decreased conception and pregnancy rates resulting in an increased risk of culling [1-4].

Bacteriological contaminations of the uterus after parturition and metabolic changes in the transition period are important etiological factors. Negative energy balance, is known to influence the number and functional properties of polymorphonuclear cells (PMN) [5,6]. There is evidence that periparturient depression of functional properties and number of PMN is of great importance for the pathogenesis of endometritis, as recently reviewed [7-9].

The identification of risk factors for endometritis will contribute to a better understanding of the underlying mechanisms in the pathogenesis of endometritis. An early identification of cows at risk for endometritis could provide new targets for intervention and the basis for changes in management practices to prevent this disease. Decreased dry matter intake (DMI) prior to parturition is associated with mobilization of lipids, which are released as non-esterified fatty acids (NEFA) from adipose tissue [10]. Decreased DMI and increased NEFA levels are associated with periparturient suppression of the immune function, resulting in a greater susceptibility of cows to infection [11]. Cows with clinical or subclinical ketosis have shown impaired phagocytic activity of PMN isolated from milk and blood [12-14]. Elevated serum BHBA concentrations in the first two weeks postpartum were indicative of an increased risk (OR = 3.35) of metritis [15], a common pre-stage for CE and SCE, and decreased pregnancy risk (OR = 0.48)[16]. A reduction of probability of pregnancy of 20 to 50% was found, depending on the magnitude and duration of elevated serum BHBA (≥1,000 μmol in wk +1 postpartum, P = 0.04 or ≥1,400 μmol in wk +2 postpartum, P = 0.01)[16]. A relationship between the plasma concentration of bilirubin and the respiratory burst activity of bovine PMN in vitro was found [17]. It was hypothesized by the authors that bilirubin has potential as a diagnostic marker of impaired neutrophil function and consequently for identification of cows at risk around parturition. Furthermore, elevated urea concentrations had an effect of decreasing uterine pH during the luteal phase [18-20]. Bovine endometrial cells in culture responded directly to increasing urea concentrations with alteration in pH gradient [21]. This effect might facilitate subsequent bacterial growth and lower local immune defense.

Few publications are available describing relationships between metabolic disorders and the prevalence of CE and SCE under field conditions. Hammon et al. [5] reported significantly elevated concentrations of NEFA and BHBA around parturition for cows with CE and SCE compared with healthy cows. Decreased PMN function and elevated plasma levels of NEFA prior to parturition and elevated plasma levels of BHBA postpartum were associated with uterine disorders later in lactation. However, predictor cut points have not been determined yet.

The objective of this study was to investigate the relationship between elevated serum concentrations of NEFA, BHBA, bilirubin and urea in the periparturient period and the prevalence of CE and SCE. Specifically, we set out to determine sensitivity and specificity of these metabolic parameters for the detection of CE and SCE, establishing optimal predictor cut points for daily practical use.

## Results

### Prevalence of endometritis

Overall prevalence of CE and SCE was 18.7% and 12.4%, respectively. Prevalence of CE and SCE at wk +5 in PP cows was 23.4% and 7.8%, respectively and in MP cows 15.9% and 15.2%, respectively.

### Primiparous cows

Serum concentrations of urea at wk +1 differed between health categories. All other metabolic parameters did not differ between health categories in PP cows (See additional file 1: Table S1 - Descriptive statistics of serum concentrations of NEFA, BHBA, bilirubin, and urea in relation to health categories for primiparous cows). Binary logistic regression revealed that in PP cows (n = 77), higher concentrations of urea at wk +1 were associated with increased odds of CE (P = 0.04) at wk +1 (Table 1). An optimal predictor cut point of 3.9 mmol/L (sensitivity 61%, specificity 70%, AUC 0.68) was determined.

**Table 1:** Logistic regression models showing the effect of metabolic parameters on the risk of health category for cows at week 5 postpartum^1^

Parity group	Metabolic parameter	**Time of Sampling**^**a**^	**Health category**^**b **^**(n)**	P value	**Odds ratio**^**c**^	95% confidence interval
Primiparous cows	Urea	+1	CE (18) vs. H (53)	0.04	1.65	1.03-2.65

	Urea	+1	CE (18) vs. SCE (6)	0.09	3.07	0.84-11.29

Multiparous cows	NEFA	-1	SCE (20) vs. H (91)	0.04	12.07	1.07-136.27

	NEFA	-1	CE (21) vs. H (91)	0.05	9.08	1.00-82.28

	Urea	+5	SCE (20) vs. CE (21)	0.03	0.49	0.26-0.93

	Urea	+5	H (91) vs. CE (21)	0.01	0.57	0.37-0.86

	NEFA	+5	SCE (20) vs. H (91)	0.06	4.09	0.93-17.95

^1 ^Only combinations were a difference (P < 0.05) between median values for NEFA, BHBA, bilirubin and urea in the health categories was found by Kruskal-Wallis-H-test are shown.

^a ^relative to calving in weeks

^b ^Health category: healthy (H), clinical endometritis (CE) and subclinical endometritis (SCE)

^c ^Odds ratios are based on the continuous variables of the logistic regression models with an 1 mmol/L increase of metabolites as units.

### Multiparous cows

Serum concentrations of NEFA at wk -1 and wk +5 and of urea at wk +5 differed between health categories. All other metabolic parameters did not differ between health categories in MP cows (See additional file 2: Table S2 - Descriptive statistics of serum concentrations of NEFA, BHBA, bilirubin, and urea in relation to health categories for multiparous cows). Higher concentrations of NEFA at wk -1 were associated with increased odds of CE (P = 0.05) and SCE (P = 0.04) at wk +5 compared with healthy cows (Table 1). An optimal predictor cut point of 0.3 mmol/L for the discrimination between healthy and CE cows (sensitivity 38%, specificity 87%, AUC 0.66) and healthy and SCE cows (sensitivity 35%, specificity 89%, AUC 0.65) was determined. Higher concentrations of urea at wk +5 were associated with decreased odds of CE compared with healthy (P = 0.01) and SCE (P = 0.03) cows (Table 1). An optimal predictor cut point of 3.8 mmol/L for the discrimination between healthy and CE cows (sensitivity 52%, specificity 81%, AUC 0.70) was determined. For the discrimination between SCE and CE cows an optimal predictor cut point of 3.5 mmol/L (sensitivity 48%, specificity 90%, AUC 0.72) was determined.

## Discussion

Previous studies demonstrated negative impacts of elevated concentrations of NEFA, BHBA, bilirubin, and urea on PMN function [5,12,13,17], uterine environment [18-20,22] and the prevalence of metritis [5,15,16,23,24]. These findings led to our hypothesis that elevated concentrations of these metabolic parameters might serve as indicators for the presence of endometritis. Differences between PP and MP cows in the regulation of fat tissue mobilization [25] and the DMI around parturition [23] have been described. Therefore, in our study metabolic profiles were analyzed separately for MP and PP cows. In MP cows, NEFA concentrations measured at wk -1 were a significant predictor for cows at higher risk of CE or SCE (CE: P = 0.05, SCE: P = 0.04) with lower concentrations in healthy cows. Sensitivities were low, but specificities were fairly high (CE: sensitivity 38%, specificity 87%; SCE: sensitivity 35%, specificity 89%). Yet, our data generated in the field support evidence from earlier in vitro studies [6,26,27] showing that high concentrations of NEFA can affect functions of bovine blood PMN. Also this study confirms previous findings [5] on an association between energy status prior to calving and uterine health in the postpartum dairy cow utilizing a different diagnostic technique (i.e. cytobrush) for SCE that has been described as more reliable than the uterine lavage [28]. To our knowledge this is the first study to describe an exact predictor cut point for NEFA to discriminate between healthy and diseased cows.

Elevated concentrations of BHBA and other ketone bodies have been shown to impair the proliferation of bone marrow cells (> 1.0 mmol/L) [14], the proliferation of lymphocytes (6.25 mmol/L) in vitro [29], the in vitro chemotactic differentials of leukocytes (> 1.6 mmol/L) [30] and the respiratory burst activity of PMN (2.5 mmol/L) [12] in cattle. Field studies found elevated concentrations of BHBA during early lactation in cows with CE and SCE compared to healthy cows [5] and elevated BHBA concentrations (> 1.2 mmol/L) in the first week postpartum indicative of an increased risk of subsequent metritis [15]. Surprisingly, our data do not confirm the diagnostic value of BHBA concentrations to predict endometritis. Differences in experimental designs, methodologies and disease definitions might have contributed to this discrepancy. In the field studies cited metritis or endometritis was diagnosed earlier in lactation (21 to 28 days postpartum [5] and before 15 days postpartum [15]) than in the current study (28 to 35 days postpartum). Furthermore, SCE was diagnosed by uterine lavage [5] and a higher threshold of PMN (> 25% vs. > 18% in our study) was used.

Alterations of uterine pH caused by elevated concentrations of urea [19,20] have been demonstrated to affect the viability of embryos [31-33]. This study investigated the relationship between serum concentrations of urea and uterine health. Multiparous cows with CE had lower (healthy vs. CE: P = 0.01, SCE vs. CE: P = 0.03) plasma concentrations of urea at wk +5. This could be explained by a possible lower feed intake of cows with CE. A recent study [23] showed that cows with uterine disease occurring in the 3 weeks postpartum consumed less dry matter during the transition period compared to healthy cows. Huzzey et al. [23], however, diagnosed metritis, not endometritis, so criteria for uterine disease were not similar to the present study. Also, it is not clear whether the reduction in DMI is the reason for uterine disease or a consequence. Information concerning the effect of a lower urea concentration, as found in cows with CE in this study, on the uterine environment or on PMN function is to our knowledge not available. On the other side, PP cows with CE had higher serum concentrations of urea at wk +1. We can only speculate about the reason for the opposing results concerning urea concentrations in PP and MP cows with CE in this study. In cows with a ruminal flora not adapted to lactational rations, the dietary protein supply exceeds the energy availability in the bovine rumen, which results in higher urea concentrations [34]. It is possible that in our study PP cows faced a more abrupt diet transition than MP cows due to the change in ration. But our study does not provide sufficient data to prove this hypothesis.

The body condition score (BCS) is used to appraise body fat in dairy cattle. The change in BCS is an indirect measurement of the fat metabolism and thereby related to the tested metabolic parameters, particularly NEFA and BHBA [35]. Measurement of the chosen metabolites, however, is more objective, repeatable, and subtle than the BCS. Furthermore, the parameters offer information about the actual differences between cows [34]. Therefore, BSC was not included in the study. We are aware, however, that the inclusion of the BCS could have added another semi-quantifiable factor of potential influence on CE and SCE.

For the determination of the optimal predictor cut points the statistical software MedCalc utilizes the Youden's index [36]. The index balances sensitivity and specificity equally. It is important to note that depending on the goals of diagnosing cows with CE or SCE one could use a different approach such as choosing a threshold based on high sensitivity or high specificity.

## Conclusions

The combinations of sensitivity and specificity for the predictors cut points determined in this study (in PP cows: urea at wk +1, in MP cows: NEFA at wk -1 and urea at wk +5) were low (35 to 61% and 70 to 90%, respectively) and unsatisfactory for practical use. Therefore, the serum concentrations of NEFA, BHBA, bilirubin or urea at wk -1, wk +1 or wk+5 relative to calving are unsatisfactory for disease prediction. This study was designed as a first approach to establish predictor cut points for daily practical use. Further research, with tighter sampling intervals around parturition, measurement of body weight, and monitoring of actual DMI, is required to better describe factors that contribute to the risk of postpartum uterine disease.

## Methods

### Study farm

The study was conducted on a commercial dairy farm in Brandenburg, Germany, housing 900 Holstein-Friesian cows. Samples sizes were calculated using a power calculator with α set at 0.05 and power set at 80% for one-tailed tests. Results from preliminary studies indicated estimated differences of means between health categories as well as standard deviations. A total of 209 cows (77 primiparous cows, 132 multiparous cows) that calved between December 2005 and June 2006 were enrolled in the study. The median age of all cows included in the study was 3 years (min. 2 years, max. 10 years). The median of the parity of cows was 2 (min. 1, max. 9). The cows were housed in a free-stall barn with slotted floors and cubicles lined with rubber mats. Herd average milk yield was 9,259 kg (4.1% fat, 3.5% protein) per cow per year. A total mixed ration containing (in % dry matter) 35.4.% corn silage, 23.1% grass silage, 8.8% rye silage, 7.8% sugar beet pulp, 6.4% rumen protected rape seed, 5.8% brewer's grain, 5.4% minerals, 3.7% rape forage cake and 3.6% soya meal, was mixed and offered twice daily after milking at about 08:00 and 16:00. Water was available ad libitum.

### Study design

Blood samples were collected from all cows in the last week prior to parturition (wk -1), the first week postpartum (wk +1) and between 28 and 35 d postpartum. Blood sampling was performed at the same time every day, i.e. within 1 h after the morning feed, by puncture of the vena coccygea mediana. A vacutainer system (Venoject II, Terumo Europe N.V., Leuven, Belgium) was used. Samples were kept at 4°C until centrifugation (10 min, 1.000 × g) within 8 h and serum was stored in two aliquots at -25°C until analysis.

Serum concentrations of NEFA, BHBA and bilirubin were measured using colometric enzymatic reactions (NEFA C Test, Wako Chemicals GmbH, Neuss, Germany; Autokit 3-HB, Wako Chemicals GmbH, Neuss, Germany and Bilirubin total (NBD) Konelab/T Series, Thermo Fisher Scientific GmbH, Dreieich, Germany, respectively) with an automated wet chemistry analyzer (Olympus AU 400, Olympus, Hamburg, Germany). Urea concentrations were measured quantitatively using the Konelab T Series instrument for chemical analysis (Urea, Thermo Scientific, Dreieich, Germany). The laboratory was previously validated using the University of Guelph (Pearson correlation r = 0.91, n = 200). All analyses were performed according to the manufacturers' instructions.

At wk +5, all cows were examined for signs of CE by external and internal inspection. Internal inspection was performed by vaginoscopy. Based on the definition of Sheldon et al. [37], CE was characterized by the presence of purulent (> 50% pus) or mucopurulent (approximately 50% pus, 50% mucus) discharge in the vagina. Cows with CE were treated intramuscularly with 0.5 mg of cloprostenol (PGF Veyx forte, Veyx Pharma GmbH, Schwarzenborn, Germany). Three weeks later the treated cows were re-examined and re-treated if not cured. If purulent or mucopurulent discharge was not found at wk +5 by vaginoscopy an endometrial cytological sample was taken using the Cytobrush technique [38]. Because the definition of a cut point for SCE is under intensive discussion [2,28,38-41] we applied two extreme cut points, 5% PMN [4] and 18% PMN [2], to our data set. The exploration of both cut points did not change the conclusion of the study. Time of examination and technique of cell collecting of this study match with methods of Kasimanickam et al. (2004), therefore, results of the 18% PMN cut point are presented here.

### Statistical analysis

Preliminary analyses indicated differences between mean metabolite concentrations between PP and MP cows, therefore, data of PP and MP cows were analyzed separately. For statistical analyses, cows were assigned to one of three categories (CE, SCE, or healthy). Differences in each blood parameter at wk -1, wk +1 and wk +5 between the health categories were analyzed using Kruskal-Wallis-H-test. Blood parameters with differences (P < 0.05) were analyzed by binary logistic regression models to assess the association between a metabolic parameter and health categories. Separate models were calculated for CE vs. healthy, SCE vs. healthy and CE vs. SCE, respectively. The metabolic parameters were included as predictors. If an association (P < 0.05) was found, receiver operating characteristic (ROC) analysis was used to determine the optimal predictor cut point (i.e. Youden's index) and corresponding sensitivity and specificity to discriminate between the two health categories. The area under the ROC curve (AUC) was used to assess the distinguishing ability of the metabolic parameter (the higher the value the better the distinguishing ability with values between 0.5 and 1.0) [42]. ROC analyses were performed by MedCalc software [43]. All other analyses were performed by using SPSS software [44].

## Authors' contributions

TBK participated in the design of the study, carried out the clinical assessments and data acquisition, performed data analyses and drafted the manuscript. MD conceived the study, participated in its design and coordination, and helped to draft the manuscript. BAT investigated the data analyses performed by TBK. WH participated in the design of the study and coordination, and helped to draft the manuscript. All authors read and approved the final manuscript.

## Supplementary Material

Additional file 1 **Table S1 - Descriptive statistics of serum concentrations of NEFA, BHBA, bilirubin and urea in relation to health categories (healthy, clinical endometritis, subclinical endometritis) for primiparous cows (n = 77)**. Table on a landscape page Click here for file

Additional file 2 **Table S2 - Descriptive statistics of serum concentrations of NEFA, BHBA, bilirubin and urea in relation to health categories (healthy, clinical endometritis, subclinical endometritis) for multiparous cows (n = 132)**. Table on a landscape page Click here for file
