# Supplementary material for: Correlations between periparturient serum concentrations of non-esterified fatty acids, beta-hydroxybutyric acid, bilirubin, and urea and the occurrence of clinical and subclinical postpartum bovine endometritis
Source: BMC Vet Res. 2010 Oct 27;6:47. doi: 10.1186/1746-6148-6-47 (PMC2988005; doi:10.1186/1746-6148-6-47)
Supplement: Additional file 1 — Table S1 - Descriptive statistics of serum concentrations of NEFA, BHBA, bilirubin and urea in relation to health categories (healthy, clinical endometritis, subclinical endometritis) for primiparous cows (n = 77). Table on a landscape page [file 1746-6148-6-47-S1.DOC]

Table S1

Descriptive statistics of serum concentrations of NEFA, BHBA, bilirubin, and urea in relation to health categories (healthy, clinical endometritis, subclinical endometritis) for primiparous cows.

|  | Weeks relative  to calving | Metabolite concentration | | | | | | | | | | | |
| --- | --- | --- | --- | --- | --- | --- | --- | --- | --- | --- | --- | --- | --- |
|  |  | Healthy  (n = 53) | | |  | Clinical endometritis  (n = 18) | | |  | Subclinical endometritis  (n = 6) | | |  |
|  |  | Median | 1^e^ quartile | 3^e^ quartile |  | Median | 1^e^ quartile | 3^e^ quartile |  | Median | 1^e^ quartile | 3^e^ quartile |  |
| NEFA (mmol/L) | -1 | 0.15 | 0.10 | 0.35 |  | 0.18 | 0.12 | 0.41 |  | 0.28 | 0.10 | 0.64 |  |
|  | +1 | 0.52 | 0.36 | 0.71 |  | 0.53 | 0.31 | 0.73 |  | 0.42 | 0.33 | 0.64 |  |
|  | +5 | 0.17 | 0.12 | 0.31 |  | 0.18 | 0.13 | 0.30 |  | 0.22 | 0.15 | 0.47 |  |
| BHBA (µmol/L) | -1 | 596 | 458 | 709 |  | 590 | 530 | 772 |  | 652 | 502 | 652 |  |
|  | +1 | 648 | 431 | 868 |  | 699 | 453 | 699 |  | 698 | 611 | 841 |  |
|  | +5 | 561 | 467 | 736 |  | 550 | 380 | 814 |  | 603 | 443 | 774 |  |
| Bilirubin (µmol/L) | -1 | 3.7 | 3.0 | 5.4 |  | 3.6 | 2.6 | 5.1 |  | 4.0 | 2.0 | 7.2 |  |
|  | +1 | 7.6 | 5.5 | 9.3 |  | 8.8 | 4.6 | 16.8 |  | 7.1 | 4.2 | 8.9 |  |
|  | +5 | 3.0 | 2.4 | 3.8 |  | 2.9 | 2.5 | 3.4 |  | 2.7 | 1.9 | 3.7 |  |
| Urea (mmol/L) | -1 | 3.3 | 2.5 | 4.0 |  | 3.5 | 3.1 | 3.9 |  | 3.4 | 2.6 | 4.6 |  |
|  | +1 | 3.2^a^ | 2.7 | 4.2 |  | 4.0^b^ | 3.2 | 5.0 |  | 3.2^a^ | 2.5 | 3.7 |  |
|  | +5 | 2.9 | 4.0 | 5.9 |  | 4.7 | 3.6 | 5.7 |  | 4.8 | 3.9 | 6.3 |  |

Within rows median values with different superscript are different (P < 0.05) revealed by Kruskal-Wallis-H-test.

Exact P values: Urea at wk +1: healthy vs. clinical endometritis: 0.023, subclinical endometritis vs. clinical endometritis: 0.047.
