# Supplementary material for: Correlations between periparturient serum concentrations of non-esterified fatty acids, beta-hydroxybutyric acid, bilirubin, and urea and the occurrence of clinical and subclinical postpartum bovine endometritis
Source: BMC Vet Res. 2010 Oct 27;6:47. doi: 10.1186/1746-6148-6-47 (PMC2988005; doi:10.1186/1746-6148-6-47)
Supplement: Additional file 2 — Table S2 - Descriptive statistics of serum concentrations of NEFA, BHBA, bilirubin and urea in relation to health categories (healthy, clinical endometritis, subclinical endometritis) for multiparous cows (n = 132). Table on a landscape page [file 1746-6148-6-47-S2.DOC]

Table S2 Descriptive statistics of serum concentrations of NEFA, BHBA, bilirubin, and urea in relation to health categories (healthy, clinical endometritis, subclinical endometritis) for multiparous cows.

|  | Weeks relative  to calving | Metabolite concentration | | | | | | | | | | | |
| --- | --- | --- | --- | --- | --- | --- | --- | --- | --- | --- | --- | --- | --- |
|  |  | Healthy  (n = 91) | | |  | Clinical endometritis  (n = 21) | | |  | Subclinical endometritis  (n = 20) | | |  |
|  |  | Median | 1^e^ quartile | 3^e^ quartile |  | Median | 1^e^ quartile | 3^e^ quartile |  | Median | 1^e^ quartile | 3^e^ quartile |  |
| NEFA (mmol/L) | -1 | 0.12^a^ | 0.09 | 0.21 |  | 0.18^b^ | 0.12 | 0.41 |  | 0.17^b^ | 0.11 | 0.47 |  |
|  | +1 | 0.55 | 0.40 | 0.76 |  | 0.43 | 0.39 | 0.71 |  | 0.67 | 0.40 | 0.75 |  |
|  | +5 | 0.20^a^ | 0.13 | 0.28 |  | 0.22^a/b^ | 0.14 | 0.35 |  | 0.30^b^ | 0.19 | 0.47 |  |
| BHBA (µmol/L) | -1 | 650 | 538 | 806 |  | 695 | 565 | 817 |  | 707 | 540 | 833 |  |
|  | +1 | 861 | 693 | 1286 |  | 864 | 679 | 1115 |  | 974 | 748 | 1562 |  |
|  | +5 | 660 | 561 | 958 |  | 658 | 529 | 1248 |  | 691 | 529 | 863 |  |
| Bilirubin (µmol/L) | -1 | 3.2 | 2.4 | 5.0 |  | 4.5 | 3.1 | 6.1 |  | 4.7 | 2.9 | 5.7 |  |
|  | +1 | 6.6 | 4.8 | 10.4 |  | 6.9 | 5.1 | 8.4 |  | 8.1 | 6.1 | 8.1 |  |
|  | +5 | 3.2 | 2.6 | 4.4 |  | 3.0 | 2.4 | 6.1 |  | 3.7 | 2.9 | 4.6 |  |
| Urea (mmol/L) | -1 | 4.2 | 3.4 | 5.3 |  | 4.8 | 3.6 | 5.2 |  | 3.8 | 3.2 | 5.0 |  |
|  | +1 | 4.1 | 3.3 | 5.2 |  | 4.5 | 4. 0 | 4.8 |  | 4.2 | 3.1 | 5.3 |  |
|  | +5 | 4.8^a^ | 4.0 | 5.7 |  | 3.8^b^ | 3.2 | 4.8 |  | 4.6^a^ | 3.9 | 5.5 |  |

Within rows median values with different superscript are different (P < 0.05) revealed by Kruskal-Wallis-H-test.

Exact P values: NEFA at wk -1: healthy vs. clinical endometritis: 0.012, healthy vs. subclinical endometritis: 0.026; NEFA at wk +5: healthy vs. subclinical endometritis: 0.034; urea at wk +5: healthy vs. clinical endometritis: 0.004, clinical endometritis vs. subclinical endometritis: 0.018.
